# Risk assessment of interstate pipelines using a fuzzy-clustering approach

**DOI:** 10.1038/s41598-022-17673-3

**Published:** 2022-08-12

**Authors:** A. Osman, M. Shehadeh

**Affiliations:** College of Engineering and Technology, Arab Academy for Science, Technology and Maritime Transport, Alexandria, Egypt

**Keywords:** Mechanical engineering, Engineering, Mathematics and computing

## Abstract

Interstate pipelines are the most efficient and feasible mean of transport for crude oil and gas within boarders. Assessing the risks of these pipelines is challenging despite the evolution of computational fuzzy inference systems (FIS). The computational intricacy increases with the dimensions of the system variables especially in the typical Takagi–Sugeno (T–S) fuzzy-model. Typically, the number of rules rises exponentially as the number of system variables increases and hence, it is unfeasible to specify the rules entirely for pipeline risk assessments. This work proposes the significance of indexing pipeline risk assessment approach that is integrated with subtractive clustering fuzzy logic to address the uncertainty of the real-world circumstances. Hypothetical data is used to setup the subtractive clustering fuzzy-model using the fundamental rules and scores of the pipeline risk assessment indexing method. An interstate crude-oil pipeline in Egypt is used as a case study to demonstrate the proposed approach.

## Introduction

Pipelines are regarded as the most secure, cost-effective, efficient, and dependable means of transporting combustible fluids^1^. Hence, pipelines would be an ideal choice for carrying significant amounts of petroleum. It is reported that in the period between 1990 to 2009, pipelines provided almost 70% of all oil transportation^2^.

Whilst most pipelines are subsurface and somewhat insulated from external intervention, they are nonetheless vulnerable to a variety of risks^3,4^, including fluid leakage^5–7^, which might have a negative impact on the environment or result in human casualties. Oil and gas firms prioritise pipeline integrity and safety in order to minimise leaks or system failures that might result in catastrophic or costly financial implications^8^. Pipe failure may never be completely prevented^3^; however, the total risk of failure can be minimized to an acceptable rate by implementing effective risk management measures^9–11^.

Oil and gas firms utilise a variety of risk assessment approaches, including as hazard and operability (HAZOP) evaluation, fault tree analysis, scenario-based analysis, and indexing methodologies^12–14^.

Limited information and insufficient data could lead to complex and unreliable pipeline risk assessment. To deal with such complexities, Zadeh developed a fuzzy logic system as a decision-making tool by processing linguistic information of such complex structures^15^, where this data is denoted as fuzzy sets inputs and the output risk values can be represented as a numerical sets or fuzzy sets with associated attribute values^16,17^. As a result, numerous academics have used fuzzy logic in risk assessment and other applications using imprecise data. Numerous methodologies based on fuzzy reasoning, such as the typical fuzzy inference system (FIS), were presented^18,19^.

A typical FIS is a method of mapping an input space to an output space using fuzzy logic; the inference system employs a collection of membership functions and rules for fuzzy reasoning of data; the fuzzy IF–THEN rules are implemented by experts; hence they are frequently referred to as fuzzy expert systems. One of the main topics to consider during the design process of a fuzzy inference system is how to decrease the overall range of included rules and their accompanying computing needs. The number of rules in a normal fuzzy system increases exponentially as the number of input variables increases. If there are n input variables and m membership functions for each variable, then constructing a comprehensive fuzzy inference system requires mn rules. The rule base becomes increasingly complex to apply as n rises. This dimensional issue is known as the "curse of dimensionality"^20^.

One of the potential solutions for this dimensional problem is a fuzzy inference system based on subtractive clustering, in which fuzzy IF–THEN rules are generated from input–output data.

Studies are concerned with the use of standard fuzzy inference systems in their applications^21^. They created a fuzzy risk matrix that might be utilised in upcoming fuzzy logic applications in various safety evaluations (e.g., LOPA). Fuzzy logic was merged with the traditional layer of protection analysis, and it was used in pipeline risk assessment to manage information fuzziness and inaccuracies^22^. Researchers suggested an integrated fuzzy logic model with relative risk score approach for pipeline risk assessment, based on expert knowledge, utilising the Mamdani algorithm to characterise the uncertainty inherent in the problem^23^. Ratnayake^24^ proposed a fuzzy inference system to reduce suboptimal function prioritizations in the functional failure risk (FFR) analysis applying an exemplary tailor-made risk matrix and the risk ranks are calculated by the suggested FIS. Further studies introduced a risk model for process operations in oil and gas facilities^25^. To eliminate the uncertainty of traditional risk-based maintenance (RBM) components, the fuzzy logic system (FLS) was introduced for risk modelling. Literature suggested a hybrid technique combining fuzzy set theory and a typical fault tree analysis of quantitative data for the crude oil tank fire and explosion (COTFE) fault tree in a fuzzy environment and to assess the likelihood of COTFE occurrence^26^. The traditional layer of protection analysis (LOPA) risk management technique was combined with fuzzy logic approach by Khalil et al.^27^ to create a cascaded fuzzy LOPA to avoid or reduce industrial accidents in natural gas facilities. Another study introduced fuzzy sets theory to fault and event tree techniques by replacing all variables with fuzzy numbers and retrieving the outcome of each using one of the defuzzification methods; this application may then be implemented in the "bow-tie" technique for accident scenario risk assessment^28^. Yuhua and Datao suggested a method for evaluating the likelihood of failure events in oil and gas transmission pipelines by integrating expert elicitation in fault tree analysis with fuzzy set theories and overcoming uncertainty and inaccuracies in some essential events^29^. Aqlan and Ali introduced lean manufacturing concepts in conjunction with fuzzy bow-tie analysis for a successful risk assessment procedure in the chemical industries, as well as to remove the uncertainties inherent in risks from standard bow-tie analyses^30^. A novel ranking approach was suggested by another study for supplier selection problem based on fuzzy inference system (FIS) to address the subjectivity of decision makers' judgments in the management of a sustainable supply chain^31^.

Literature recommends the use of risk scores (which might be available as tables, equations and charts) to predict the risk in several daily life aspects^32–37^. Hence, predictive tools are implemented to evaluate risks for proper decision-making. However, these tools present critical limitations^38,39^. The common risk assessment tools are diversely represented which does not facilitate their integration/combination. These representations are not adequate to cope with missing risk factors and cannot incorporate additional knowledge/information. Hence, a common representation must be simple, interpretable, flexible to incorporate additional variables and to swiftly allow several models-integration^39–44^. Therefore, this study aims to minimize these limitations through a fuzzy clustering approach to improve the performance of the risk assessment, extract information provided by the risk assessment tools, to allow new risk factor incorporations, to deal with missing risk factors, and to assure the interpretability of the model.

The notion of fuzzy logic is used in this study to evaluate the risks of a pipeline. A variety of models are developed for a pipeline section's Index Sum and Leak Impact Factor. The generated models' performance is compared to the hypothetical computed data, and the best fit model is selected using performance assessment indices such as training root mean square error (Training RMSE), check root-mean-square error (Check RMSE), and correlation coefficient (R^2^).

## Traditional indexing method

A subjective evaluation tool for assessing pipeline risks based on a combination of statistical failure data and operator experience, in which the pipeline is divided into segments based on factors such as population, land type, soil condition, coating condition, pipeline age, or any other factors determined by the evaluator.

This approach makes multiple hypotheses, including that all risks are independent and additive, that the worst-case scenario for the pipeline section is assigned, that all point values are relative rather than absolute, that the relative importance of each item is based on expert evaluations, that only risks to the public are considered, and that no consideration is given to pipeline operators or contractors.

**Figure 1:**
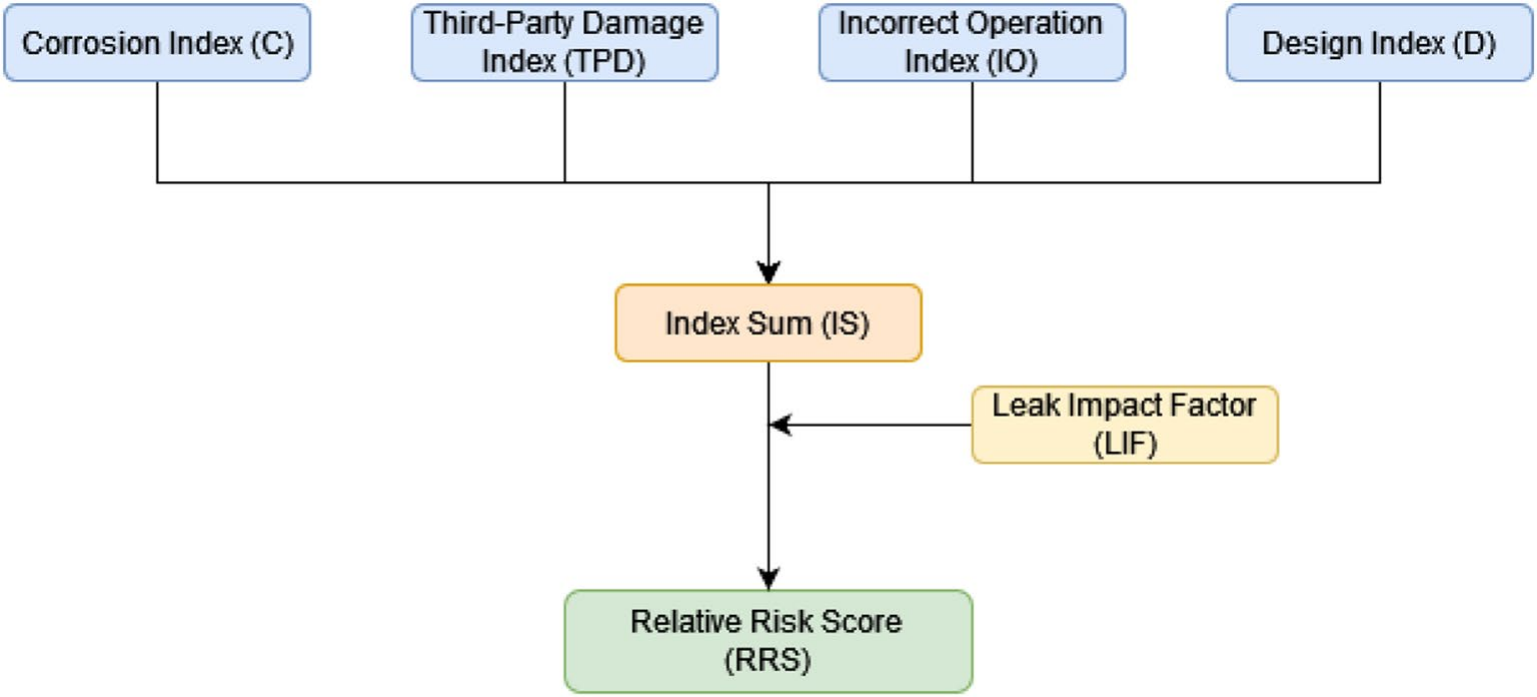
The basic risk assessment model.

Data is obtained to create an index for each type of pipeline failure initiation, including (a) third-party damage, (b) corrosion, (c) design, and (d) incorrect operations, Fig. 1 shows the basic risk assessment model. These four indices rank the likelihood and significance of all elements that maximize or minimize the likelihood of a pipeline failure. The indices are then added together to get the Index Sum, as stated in Eq. (1). As the index sum score increases, so does the probability of risk, and vice versa. The evaluation concludes with a discussion of the effects of a pipeline system breakdown. The leak impact factor is a consequence factor that is used to change the index total scores to reflect the repercussions of failure, with a greater point representing a bigger risk. The leak impact factor is the sum of the product risks (acute + chronic), leak volume, receptors, and dispersion factor, as stated in Eq. (2), where the dispersion factor equals the leak volume spill score (LV) divided by the receptors population score (RE), as indicated in Eq. (3). As demonstrated in Eq. (4), the relative risk score RRS is equal to the Index Sum (IS) divided by the Leak Impact Factor (LIF)^45^.

1 $${\text{IS}} = {\text{TPD}} + {\text{C}} + {\text{D}} + {\text{IO}}$$

2 $${\text{DF = LV / RE}}$$

3 $${\text{LIF}} = {\text{LV}} \times {\text{RE}} \times {\text{DF}} \times {\text{PH}}$$

4 $${\text{RRS = IS / LIF}}$$

## Fuzzy inference system

**Figure 2:**
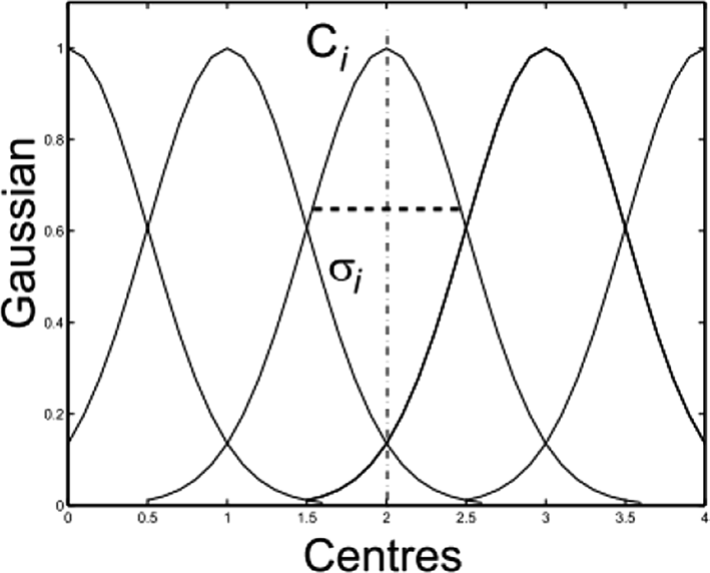
Gaussian membership functions^47^.

The fundamental principle of fuzzy set theory was introduced by Zadeh (1965)^4^ to resolve uncertainty in real-life circumstances. Fuzzy logic is used to solve issues with unsharp boundaries where membership is determined by degree. A fuzzy set defined on a universe of discourse (U) is a characterized by a membership function $$\mu (x)$$$$(x)$$, that accepts values from the interval [0, 1], where 0 indicates non-membership and 1 indicates full membership. A membership function quantifies the degree to which an element in U is similar to the fuzzy subset. For certain linguistic variables, fuzzy sets are defined. Each linguistic term can be expressed by a membership function of triangular, trapezoidal, or Gaussian form. The selection of membership function is mostly determined by variable features, accessible information, and expert opinion^26^. In this work Gaussian membership functions are employed for being the most natural^22^, smooth and nonzero at all points^46^. As a result, it can tackle real challenges with uncertain and vague data as in risk assessment studies. Gaussian membership function can be represented as illustrated in Eq. (5).

5 $$\mu_{{A^{i} }} (x) = \exp \left( { - \frac{{(c_{i} - x)^{2} }}{{2\sigma_{i}^{2} }}} \right)$$

where $c_{i}$ and ${\upsigma }_{\mathrm{i}}$ $\sigma_{i}$ are the center and width of the ith fuzzy set $A^{i}$, respectively, as shown in Fig. 2.

**Figure 3:**
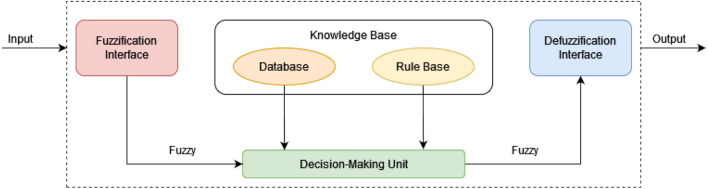
Fuzzy inference structure.

A fuzzy inference system maps an input space (universe of discourse) to an output space using fuzzy logic. A list of IF–THEN rules, membership functions that describe how each point in the input space is translated to a degree of membership between 0 and 1, and fuzzy logic operators that link with the fuzzy sets are the primary mechanisms for achieving this. As shown in the Fig. 3, a fuzzy inference system consists of: (1) knowledge base, (2) inference or decision-making unit, (3) fuzzification interface, and (4) defuzzification interface.

Several fuzzy inference models are applied in numerous applications, such as Mamdani, Takagi–Sugeno, and Tsukamoto fuzzy model. The Takagi–Sugeno and Mamdani approaches are commonly used to model real-world situations. In many ways, the two techniques are very similar to one another. The first two steps of the fuzzy inference process, fuzzification of inputs and application of fuzzy operators, are identical. The primary distinction is that Takagi–Sugeno output membership functions are either linear or constant. The Takagi–Sugeno approach is applied in this study to assess potential pipeline risks.

**Figure 4:**
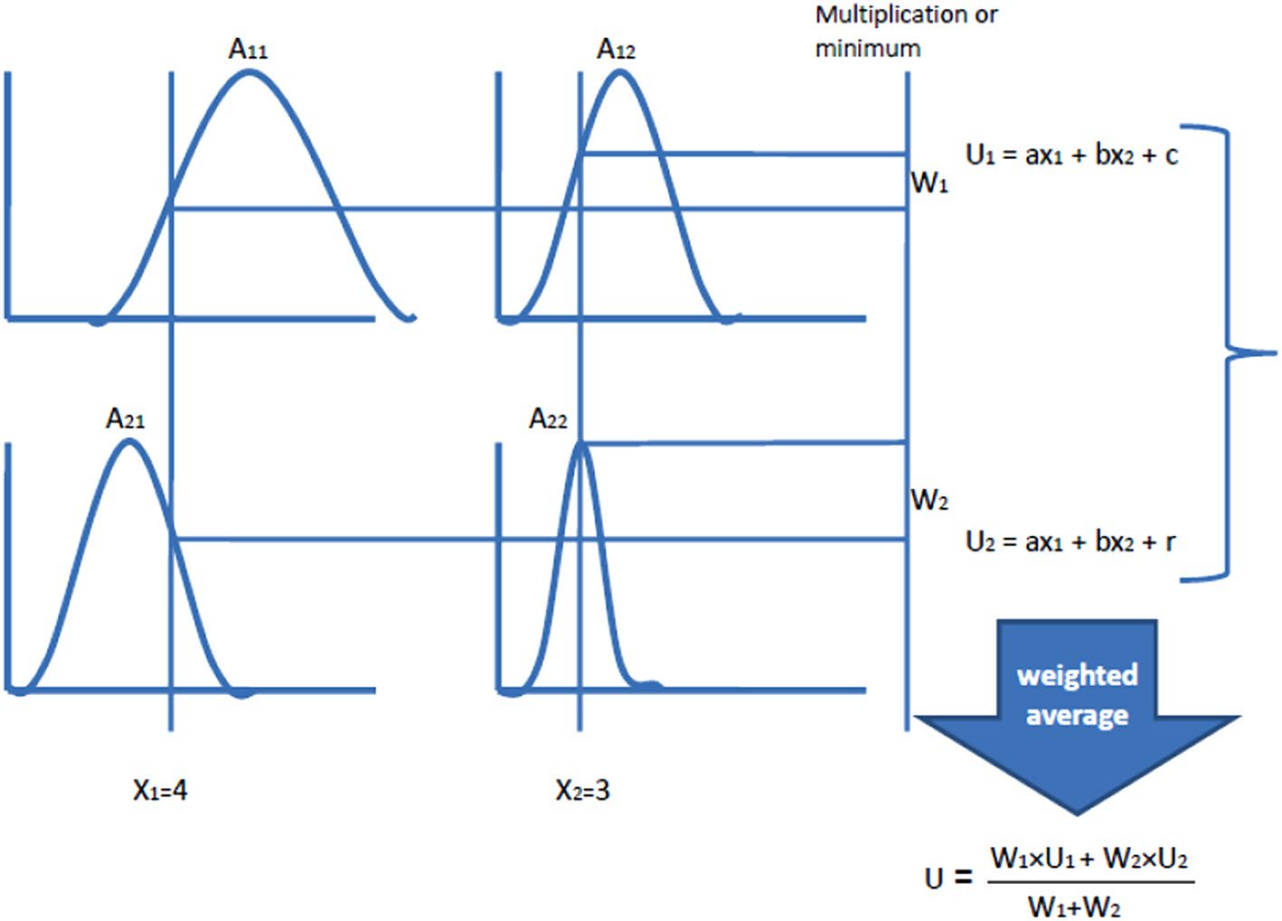
A typical TS inference mechanism for two input variables.

The TS model introduced by Takagi and Sugeno in 1985 where its major feature is the linearization of each fuzzy rule as a linear subsystem, which is utilised to simulate complicated nonlinear systems^48^. The output is a mix of all of these linear subsystems, which is accomplished by rule aggregation. The TS fuzzy model can deal with any nonlinear system with high precision and has been accepted as a universal approximator of any smooth nonlinear system^49,50^. TS rules use functions of input variables as the rule output (consequent). The general form of TS rule model having two inputs x_1_ and x_2_, and output U is as follows:

$${\text{if}}\;{\text{ x}}_{{1}} \, \;{\text{is }}\;{\text{A}}_{{1}} \, \;{\text{and }}\;{\text{x}}_{{2}} \, \;{\text{is }}\;{\text{A}}_{{2}} \, \;{\text{THEN }}\;{\text{U }}\;{\text{is }}\;{\text{z}} = {\text{f(x}}_{{1}} {\text{,x}}_{{2}} {)}$$

where z = f(x_1_, x_2_) is a crisp function of the output; A_1_ and A_2_ are linguistic terms. Figure 4 depicts a typical TS inference mechanism for two input variables.

This function is most typically linear, with fuzzy rules created linearly from input–output data, although nonlinear functions are used by adaptive approaches^51^.

The aforementioned section discusses the presence of four variables for the Index Sum (IS) model which are C, TPD, IO, and D. The fuzzy IF–THEN rules of this model can be defined as follows:

$$\begin{aligned} If \, & \left( {C \, \;is\; \, ..} \right), \, AND\; \, \left( {TPD \, \;is \, ..} \right), \, AND\; \, \left( {IO \, \;is \, ..} \right), \, AND \, \;\left( {D \, \;is \, ..} \right) \\ & THEN \, \;\left( {IS \, = \, a \times C \, + \, b \times TPD \, + \, c \times IO \, + \, d \times D \, + \, e} \right) \\ \end{aligned}$$

However, the four variables for the Leak Impact Factor (LIF) model which are PH, DF, LV, and RE. The fuzzy IF–THEN rules of this model can be defined as follows:

$$\begin{aligned} If \, & \left( {PH \, \;is \, ..} \right), \, AND\; \, \left( {LV \, \;is \, ..} \right), \, AND \, \left( {RE\; \, is \, ..} \right), \, AND \, \left( {DF\; \, is \, ..} \right) \\ & THEN \, \left( {LIF \, = \, f \times PH \, + \, g \times LV \, + \, h \times RE \, + \, i \times DF \, + \, j} \right) \\ \end{aligned}$$

The parameters a, b, c, d, and e are estimated from the training dataset of the IS model, and the parameters f, g, h, i, and j are estimated from the training dataset of the LIF model. The final output of the two fuzzy models is the weighted average of all rule outputs in each model, computed as:

6 $${\text{Final }}\;{\text{Output = }}\frac{{\sum\nolimits_{{{\text{i}} = {1}}}^{{\text{N}}} {w_{i} z_{i} } }}{{\sum\nolimits_{{{\text{i}} = {1}}}^{{\text{N}}} {w_{i} } }}$$

where N is the number of rules, $w_{i}$ is the firing strength to weight the ith fuzzy rule defined as:

7 $$w_{i} = \prod\limits_{j = 1}^{n} {\mu (A_{i}^{j} } )$$

where $n$ is the number of input variables;$\mu (A_{i}^{j} )$ is the grade of the membership function $A_{i}^{j}$.

## Research methodology

The risk assessment of pipelines could be qualitatively modelled using the expert's knowledge of the system, which is accomplished through mathematical modelling from the expert's knowledge, which includes the system's input and output data. The fuzzy clustering method is a powerful identification tool for such systems that contain potential uncertainty by grouping the input–output data into fuzzy clusters and then translating these clusters into fuzzy IF–THEN rules. This prevents identifying all of the rules as performed in conventional fuzzy inference methods. There are several fuzzy clustering methods, the most common of which is fuzzy C-means (FCM) clustering^52,53^, mountain clustering^54^, and subtractive clustering^55,56^.

Subtractive clustering method is used to conduct this research. This method, like the mountain clustering method, can auto-generate the number and initial location of cluster centres using search techniques, whereas fuzzy C-means clustering requires prior knowledge of the number of clusters. Another advantage of subtractive clustering over mountain clustering is that each data point is treated as a potential cluster centre, whereas mountain clustering treats each grid point as a potential cluster centre^57^.

Professional suggestions from previous studies^33–45^ are used to obtain data for the (IS) and (LIF) models. TPD, C, D, and IO are the input parameters of (IS). While the (LIF) input's parameters are PH, LV, DF, and RE. The two models presented in this paper use a set of statical data that consists of 625 input/output data points, a portion of which is shown in Table 1 for the (IS) model and Table 2 for the (LIF) model.

**Table 1:** Statical description on data set of IS model.

No.	C	TPD	D	IO	IS
1	0	0	0	0	0
2	0	0	0	25	25
3	0	0	0	50	50
4	0	0	0	75	75
5	0	0	0	100	100
6	0	0	25	0	25
7	0	0	25	25	50
8	0	0	25	50	75
9	0	0	25	75	100
10	0	0	25	100	125
11	0	0	50	0	50
12	0	0	50	25	75
13	0	0	50	50	100
14	0	0	50	75	125
15	0	0	50	100	150
16	0	0	75	0	75
17	0	0	75	25	100
18	0	0	75	50	125
19	0	0	75	75	150
20	0	0	75	100	175
21	0	0	100	0	100
22	0	0	100	25	125
23	0	0	100	50	150
24	0	0	100	75	175
25	0	0	100	100	200
					
625	100	100	100	100	400

**Table 2:** Statical description on data set of LIF model.

No.	LV	RE	DF	PH	LIF
1	1	1	0.25	0	0
2	1	1	0.25	5.5	1.375
3	1	1	0.25	11	2.75
4	1	1	0.25	16.5	4.125
5	1	1	0.25	22	5.5
6	1	1	1.688	0	0
7	1	1	1.688	5.5	9.284
8	1	1	1.688	11	18.56
9	1	1	1.688	16.5	27.85
10	1	1	1.688	22	37.13
11	1	1	3.125	0	0
12	1	1	3.125	5.5	17.18
13	1	1	3.125	11	34.37
14	1	1	3.125	16.5	51.56
15	1	1	3.125	22	68.75
16	1	1	4.563	0	0
17	1	1	4.563	5.5	25.09
18	1	1	4.563	11	50.19
19	1	1	4.563	16.5	76.28
20	1	1	4.563	22	100.3
21	1	1	6	0	0
22	1	1	6	5.5	33
23	1	1	6	11	66
24	1	1	6	16.5	99
25	1	1	6	22	132
					
625	6	4	6	22	3168

## Performance evaluation indices

Two different indices, including root mean square error (RMSE) and correlation coefficient (R^2^), are used to compare the outputs estimated by the established model with the expert's data output to evaluate the performance of each model. The following equations are used to compute these indices:

8 $${\text{RMSE }} = \, \sqrt {\frac{{\sum\nolimits_{{{\text{i}} = {1}}}^{{\text{N}}} {{(}A_{{\text{i}}} - P_{i} )^{2} } }}{N}}$$

9 $${\text{R}}^{{2}} = 1 - \frac{{\sum\nolimits_{{{\text{i}} = {1}}}^{{\text{N}}} {{(}A_{{\text{i}}} - P_{i} )^{2} } }}{{\sum\nolimits_{{{\text{i}} = {1}}}^{{\text{N}}} {{(}A_{{\text{i}}} - \overline{A}_{i} )^{2} } }}$$

where $P_{i}$ is the predicted values, $A_{{\text{i}}}$ is the qualitative expert's values, $\overline{A}_{i}$ is the average of the observed set, and $N$ is the number of data set.

The RMSE index, which is one of the most commonly used indices in performance evaluations, could clarify the difference between the model output and the actual value. The root mean square error (RMSE) is a non-negative number that can be zero when the predicted output exactly matches the recorded output and has no upper bound.

R^2^ is a positive number that indicates how much of the variability in dependent variable can be explained by independent variable(s) and how well the model fits the data. ${R}^{2}$ can take values between 0 and 1; which 1 indicates the model can acquire all the variability of the output variable, while 0 expresses that there is a poor correlation between model output and actual output.

**Figure 5:**
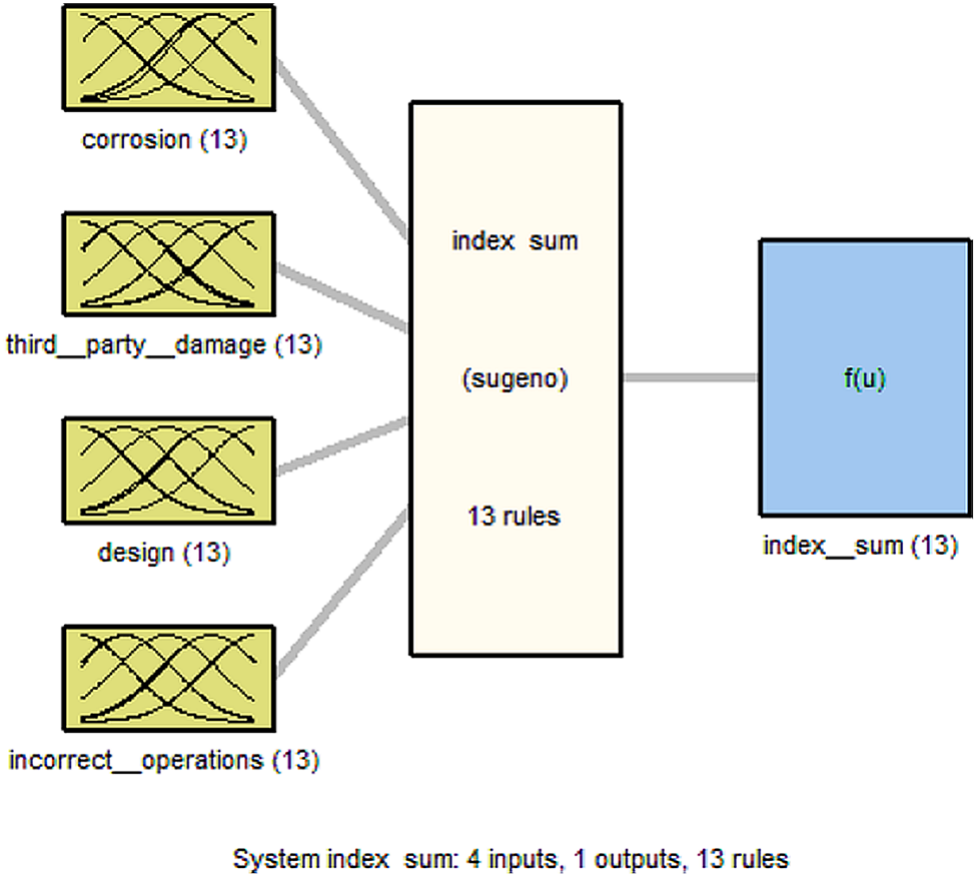
Index sum fuzzy inference structure.

**Figure 6:**
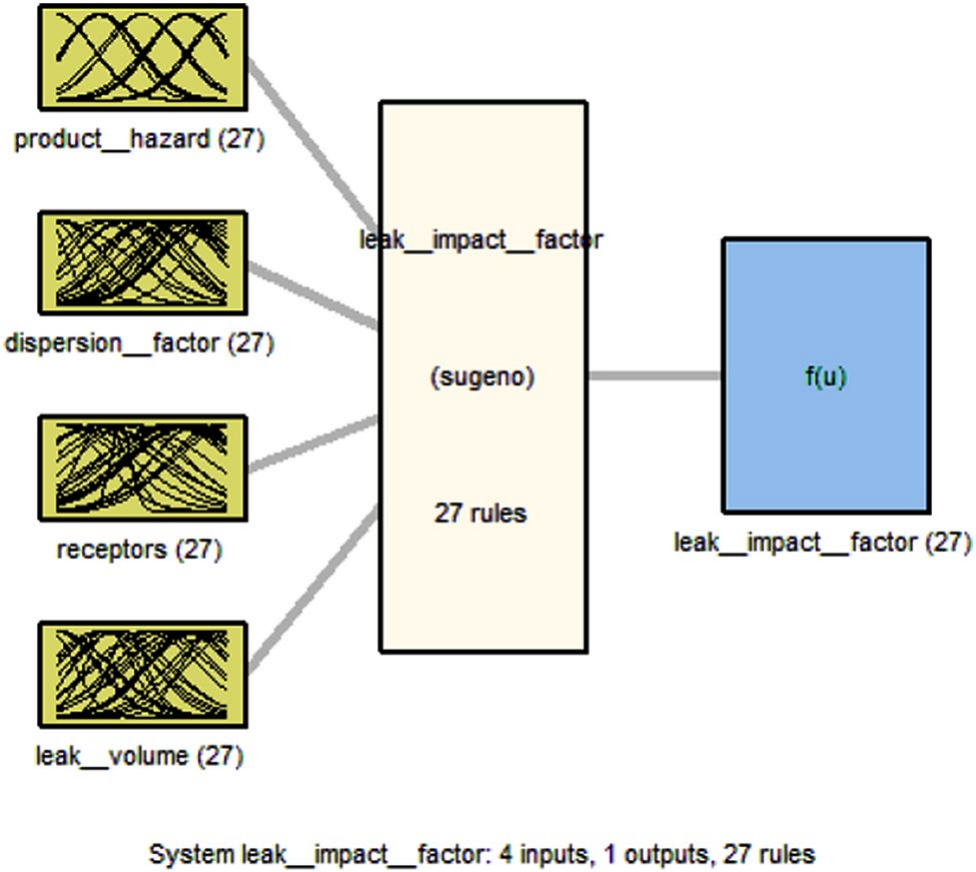
Leak impact factor fuzzy inference structure.

As shown in Figs. 5 and 6, for each model's four inputs in the index sum and the leak impact factor model, there is a single output reflecting the risk determined by expert knowledge. As shown in Tables 1 and 2, out of 625 pipeline data for index sum and leak impact factor, 500 pipeline data are used for training, i.e., to form the membership functions and produce the fuzzy IF–THEN rules; 125 pipeline data are used for testing and checking the fuzzy model established in each model to validate the model and prevent overfitting that may occur on the training data set.

MATLAB software is used to perform subtractive clustering on the pipeline index sum and leak impact factor data. In each model, the algorithm is repeated for cluster radii 0.1 through 0.9. The best fitted model in the index sum and leak impact factor models based on the best performance indices with the testing dataset has a cluster radius of 0.8 and 0.6, respectively, after applying the subtractive clustering method to the training data of the index sum and the leak impact factor with different ranges of cluster radius, as shown in Tables 3 and 4. The index sum and leak impact factor models generate 13 and 27 fuzzy rules, respectively. The established index sum model has 65 linear and 104 nonlinear parameters, whereas the established leak impact factor model has 295 linear and 472 nonlinear parameters. As shown in Tables 3 and 4, a small cluster radius generates a large number of rules and vice versa.

**Table 3:** Index sum's comparative test results of cluster radius value from 0.1 to 0.9 with selected best fitted model.

Cluster radius	Epoch number	Number of fuzzy rules	Training RMSE	Check RMSE	Correlation coefficient R^2^
0.1	4	485	5.9785 × 10^–4^	5.2103	0.9247
0.2	17	485	16.000 × 10^–4^	5.2161	0.9646
0.3	5	379	6.6872 × 10^–4^	5.8668	0.9992
0.4	2	127	2.8187 × 10^–5^	0.5839	1
0.5	12	54	1.4893 × 10^–6^	2.4321 × 10^–6^	1
0.6	9	34	3.8899 × 10^–7^	4.2578 × 10^–7^	1
0.7	18	20	1.5416 × 10^–7^	1.5269 × 10^–7^	1
**0.8**	**185**	**13**	**5.9653 × 10**^**–8**^	**7.3541 × 10**^**–8**^	**1**
0.9	2	10	5.2631 × 10^–8^	4.1280 × 10^–8^	1

Bold values are illustrated later within the following section.

**Table 4:** Leak impact factor's comparative test results of cluster radius value from 0.1 to 0.9 with selected best fitted model.

Cluster radius	Epoch number	Number of fuzzy rules	Training RMSE	Check RMSE	Correlation coefficient R^2^
0.1	1	483	2.5801 × 10^–5^	66.7306	0.7842
0.2	2	483	7.6480 × 10^–5^	66.4401	0.9052
0.3	11	339	5.8455 × 10^–5^	57.9682	0.9421
0.4	82	134	1.7843 × 10^–4^	93.7299	0.9368
0.5	78	59	1.1521	27.6552	0.9318
**0.6**	**200**	**27**	**1.4065**	**8.78140**	**0.9601**
0.7	200	17	4.4991	10.5146	0.8734
0.8	200	12	10.952	20.8779	− 0.9539
0.9	200	10	6.2846	13.0260	− 0.9044

Bold values are illustrated later within the following section.

The model performance indices, training RMSE and testing RMSE and the correlation coefficient (R^2^) for the best model of index sum (cluster radius = 0.8) obtained are 5.9653 × 10^–8^ and 7.35411 × 10^–8^ and 1 respectively. The model performance indices, training RMSE and testing RMSE and the correlation coefficient (R^2^) for the best model of leak impact factor (cluster radius = 0.6) are 1.4065 and 8.7814 and 0.9601 respectively.

The interdependence of input and output parameters derived from subtractive clustering rules can be demonstrated using control surfaces, as shown in Figs. 9 and 10 for index sum and leak impact factor, respectively.

**Figure 7:**
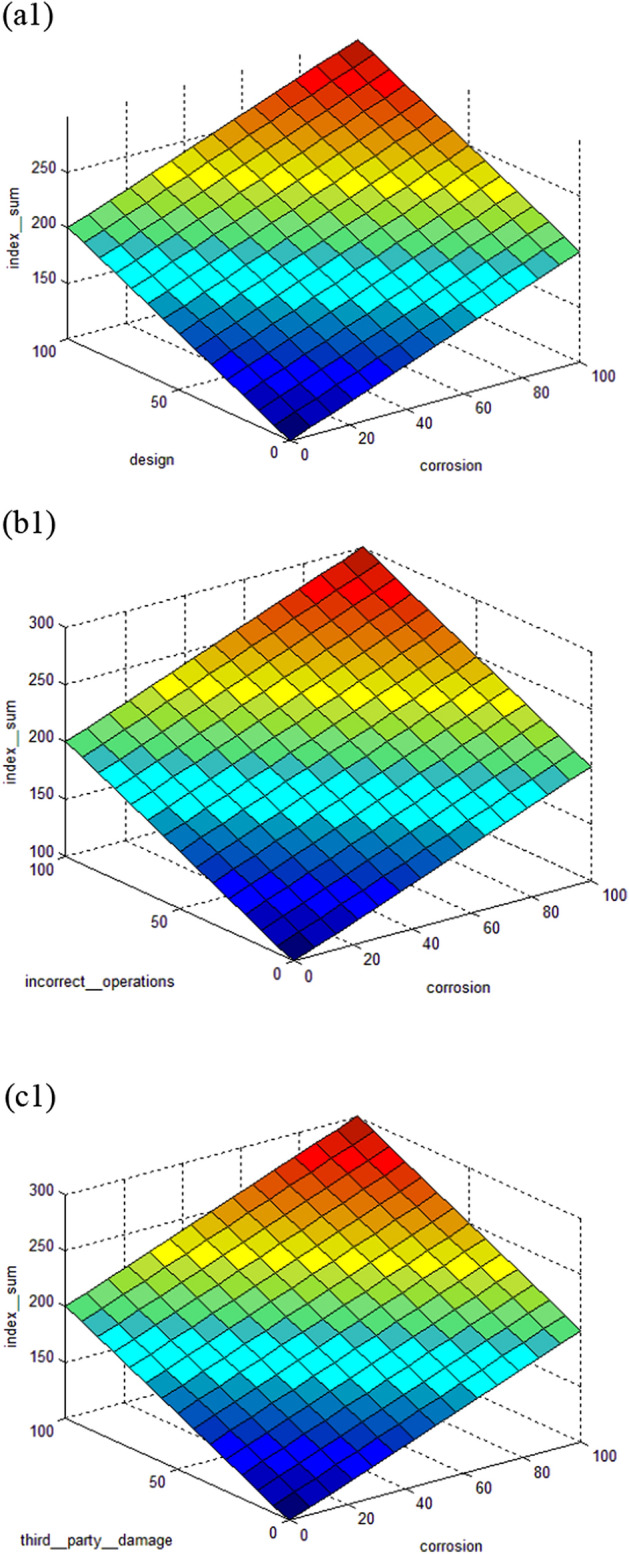
Control surface of index sum on (**a1**) design and corrosion; (**b1**) incorrect operations and corrosion; (**c1**) third party damage and corrosion.

**Figure 8:**
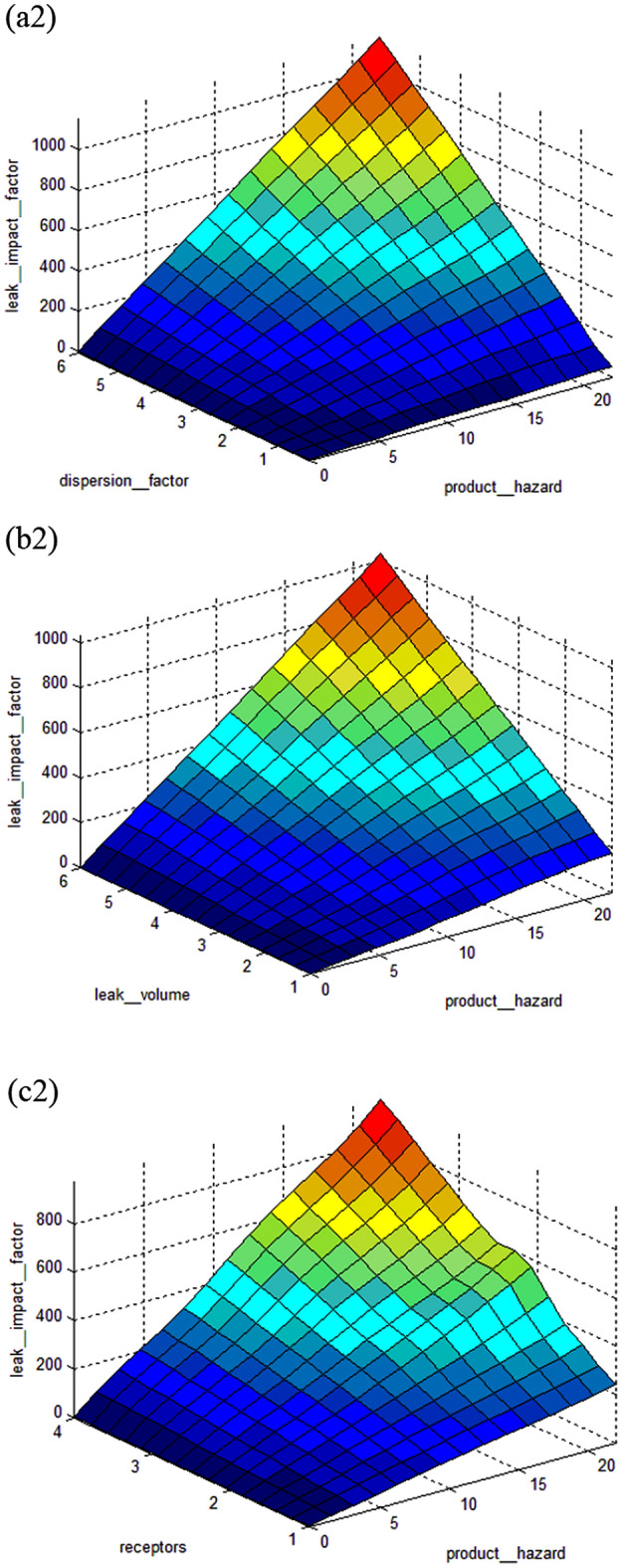
Control surface of leak impact factor on (**a2**) dispersion factor and product hazard; (**b2**) leak volume and product hazard; (**c2**) receptors and product hazard.

The index sum model is shown in Fig. 7. Figure 7(a1) indicates the interdependence of index sum on design and corrosion, Fig. 7(b1) shows the interdependence of index sum on incorrect operations and corrosion, and Fig. 7(c1) depicts the interdependence of index sum on third party damage and corrosion. While Fig. 8 of the leak impact factor model represents the interdependence of the leak impact factor on the dispersion factor and the product hazard on Fig. 8(a2) and (b2) demonstrates the interdependence of the leak impact factor on the leak volume and the product hazard, and Fig. 8(c2) depicts the interdependence of the leak impact factor on the receptors and the product hazard.

## Case study

**Figure 9:**
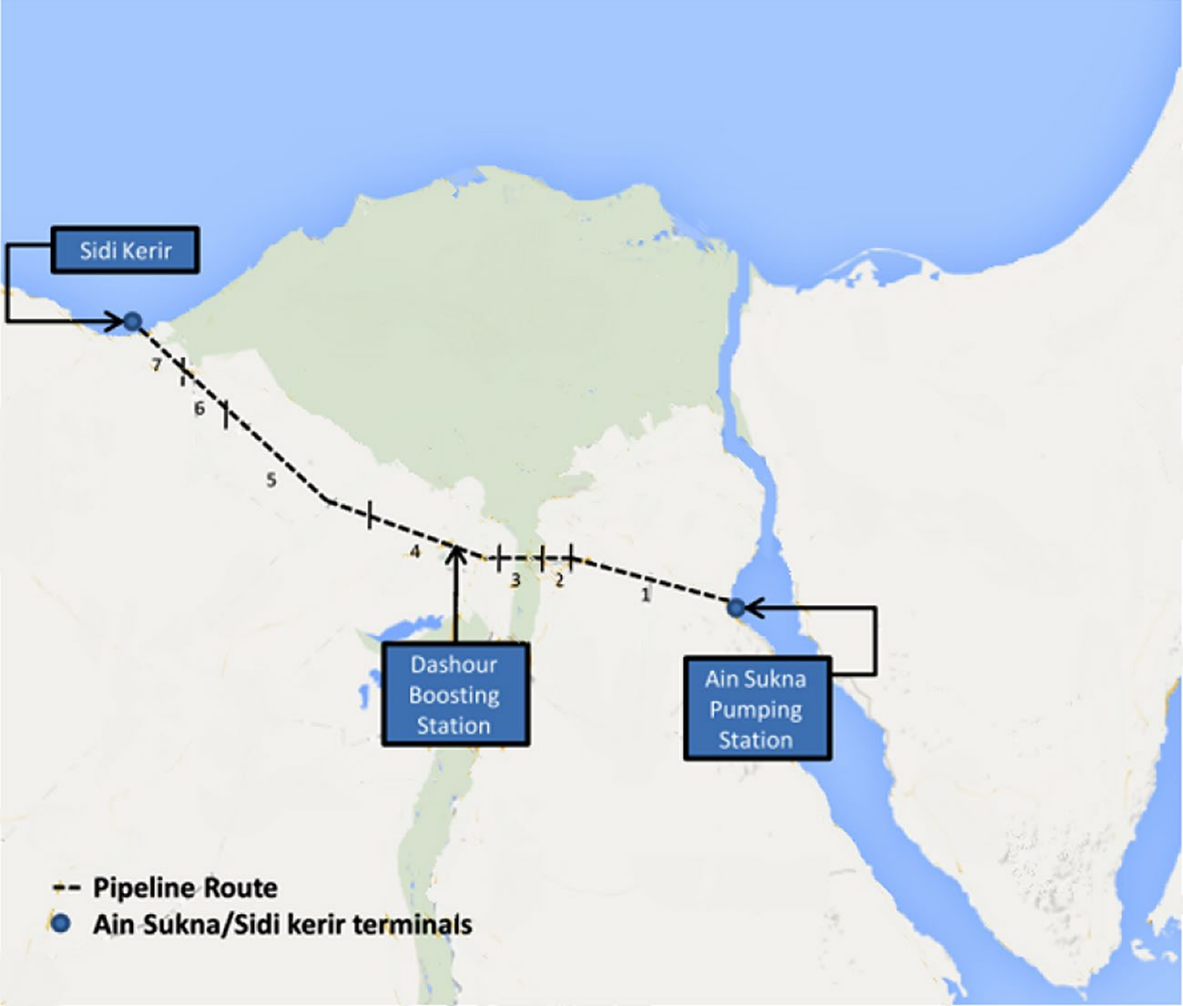
SUMED pipeline sections^59,60^.

SUMED pipeline is a typical case study used here to demonstrate the proposed pipeline risk assessment approach, Fig. 9. Since, the SUMED pipeline is critical for the international energy market because it allows for the transport of exported crude oil, which is transported by very large crude carriers VLCCs from Gulf countries and passing through the Suez Canal on their way to Europe and/or the United States^58–60^. These large tankers cannot pass through the Suez Canal fully loaded because their draught exceeds the Canal's depth. The loaded tankers are moored to a single point mooring system (SPM) at the Ain Sukhna terminal before passing through the Canal. The crude oil is then discharged from the tanker via the SPM piping system to the pipeline. Tankers can then pass through the Canal in ballast with a low draught. Crude oil is transported through two parallel pipelines, 42 inches in diameter and 320 kms long, running from the Ain Sukhna terminal to the Sidi Kerir terminal south of Cairo, where a pressure relief station protects the pipeline from overpressure^58,60^. An intermediate boosting station, comprised of six gas turbine-powered pumps, is located midway at Dahshour to assist in pushing the oil to its final destination, the Sidi Kerir terminal. After passing through the canal in ballast, the tankers are moored to a single point mooring system (SPM) at the Sidi Kerir terminal, where the oil is reloaded via terminal pumps and the SPM piping system^59,61–63^.

The pipeline's wall thicknesses range from 11.13 mm to 22.22 mm depending on the design^58^. The 320 km pipeline will be divided into seven sections of varying lengths. The pipeline is sectioned by taking the following factors into account: the type of land, soil condition, atmospheric type, population density, crossing rivers and waterways, high/low lands, and the presence of Right of Way (ROW). Sections will have the following distances and characteristics, as shown in Table 5^6,58^.

**Table 5:** Pipeline sections^58^.

Section number	Starts-ends	Pipeline length	Characteristics
1	0 km–100 km	100 km	Starts from Ain Sukna, lowest point on land, desert area
2	100 km–105 km	5 km	Passing near a cement factory, high population density
3	105 km–115 km	10 km	Passing through the river Nile, no ROW, presence of seasonal corps, high population density
4	115 km–165 km	50 km	Moderate population density
5	165 km–265 km	100 km	Low population density
6	265 km–295 km	30 km	Presence of seasonal corps, no ROW
7	295 km–320 km	25 km	Ends at Sedi Kerir, passing through lake, high population density

The risk assessment is performed on each pipeline section separately using the traditional method and the proposed model, and the results of both methods are compared in the following section of the paper. The pipeline section with the lowest RRS value is chosen as the riskiest section, which may assist pipeline operators in beginning to manage the risk on the lowest score pipeline section. To improve the reliability and safety of the lowest RRS value section, the operator may begin with the lowest scored index., e.g., low scored design index. The results of the traditional RRS method for risk assessment of 7 sections are calculated, based on Eqs. (1), (2), (3), and (4). An example is presented as follows:

$${\mathrm{IS}}_{(\mathrm{section}1)} = 84 + 83 + 1 + 82 = 250$$

$${\mathrm{DF}}_{(\mathrm{section}1)} = 2/2 = 1$$

$${\mathrm{LIF}}_{(\mathrm{section}1)} = 9\times 2\times 1\times 2 = 36$$

$${\mathrm{RRS}}_{(\mathrm{section}1)} = \frac{{\mathrm{IS}}_{(\mathrm{section}1)}}{{\mathrm{LIF}}_{(\mathrm{section}1)}}= 250/36 = 6.94$$

## Results and discussions

Table 6 displays the proposed model's output relative risk score RRS results (index sum and leak impact factor). Including index sum entry values, third-party damage, corrosion, design, and incorrect operations. And the leak impact factor, product hazard, leak volume, dispersion factor, and receptors entry values.

**Table 6:** Output RRS results of the proposed model.

Section no.	TPD	C	D	IO	PH	LV	DI	RE	IS	LIF	RRS	Rank
1	84	83	1	82	9	2	1	2	250	33.7	7.418	4
2	77	81	30	82	9	2	0.6	3	270	35.2	7.670	5
3	68	72.5	32	87	9	3	0.8	4	260	84.9	3.062	1
4	77	84	36.5	87	9	2	0.6	3	285	35.2	8.096	6
5	84	83	36.5	82	9	2	1	2	286	33.7	8.486	7
6	64	81.5	36.5	82	9	3	1.5	2	264	76.8	3.437	3
7	63	70	37	84	9	3	1.5	2	254	76.8	3.307	2

Table 6 demonstrates that Sect. 3 of the pipeline represents the lowest RRS value and ranked as the riskiest part of the pipeline as it passes through the river Nile. Section 3 is the starting point in risk management to decrease the risks on it. The risk assessor can start by enhancing the design index record of this section as it has the lowest value between the index sum indices.

The design index record can be enhanced by doing the following:

- Increase pipe safety factor.
- Increase system safety factor.
- Avoid fatigue.
- Avoid surge potential.
- Make a system hydrotest to ensure pipeline integrity.
- Avoid pipe movements.

**Figure 10:**
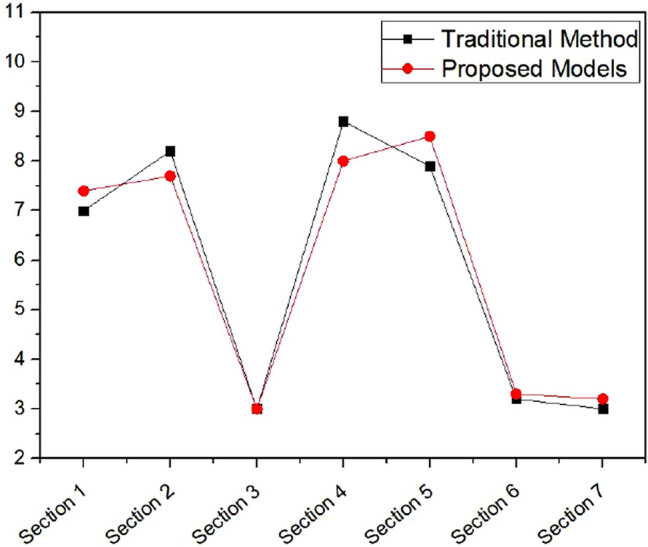
RRS results of traditional method versus proposed models.

To compare the output RRS results of the proposed model with those of the traditional method, Table 7 displays the RRS output values and ranks in both methods. Figure 10 depicts the relationship between the traditional method output RRS values and the proposed model output RRS values. The results show that the proposed fuzzy model based on subtractive clustering is an effective tool for assessing pipeline risk.

**Table 7:** Output RRS results of traditional method and proposed model.

Section number	Traditional method						Proposed model					
	IS	Rank	LIF	Rank	RRS	Rank	IS	Rank	LIF	Rank	RRS	Rank
1	250	7	36	2	6.94	4	250	7	33.7	4	7.418	4
2	270	3	32.4	3	8.30	6	270	3	35.2	3	7.670	5
3	259.5	5	81	1	3.20	2	260	5	84.9	1	3.062	1
4	284.5	2	32.4	3	8.78	7	285	2	35.2	3	8.096	6
5	286	1	36	2	7.93	5	286	1	33.7	4	8.486	7
6	264	4	81	1	3.26	3	264	4	76.8	2	3.437	3
7	254	6	81	1	3.14	1	254	6	76.8	2	3.307	2

To investigate the relationship between the qualitative method and the proposed model further, the degree of correlation between the index sum and leak impact factor obtained by the proposed subtractive clustering fuzzy model and those obtained by the qualitative method was calculated as:

10 $$\rho = \frac{{{\text{cov}} (x,y)}}{{\sigma_{x} \sigma_{y} }} = \frac{{\sum\nolimits_{i = 1}^{n} {(x_{i} - \overline{x})(y_{i} - \overline{y})} }}{{\sqrt {\sum\nolimits_{i = 1}^{n} {(x_{i} - \overline{x})^{2} } } \sqrt {\sum\nolimits_{i = 1}^{n} {(y_{i} - \overline{y})^{2} } } }}$$

where, $x$ = qualitative output results, $y$ = fuzzy inference output results, $\overline{x}$ = mean value of $x$, $\overline{y}$ = mean value of $y$, ${\text{cov}} (x,y)$ = covariance of $x$ and $y$, $\sigma_{x}$ = standard deviation of $x$, $\sigma_{y}$ = standard deviation of $y$.

**Figure 11:**
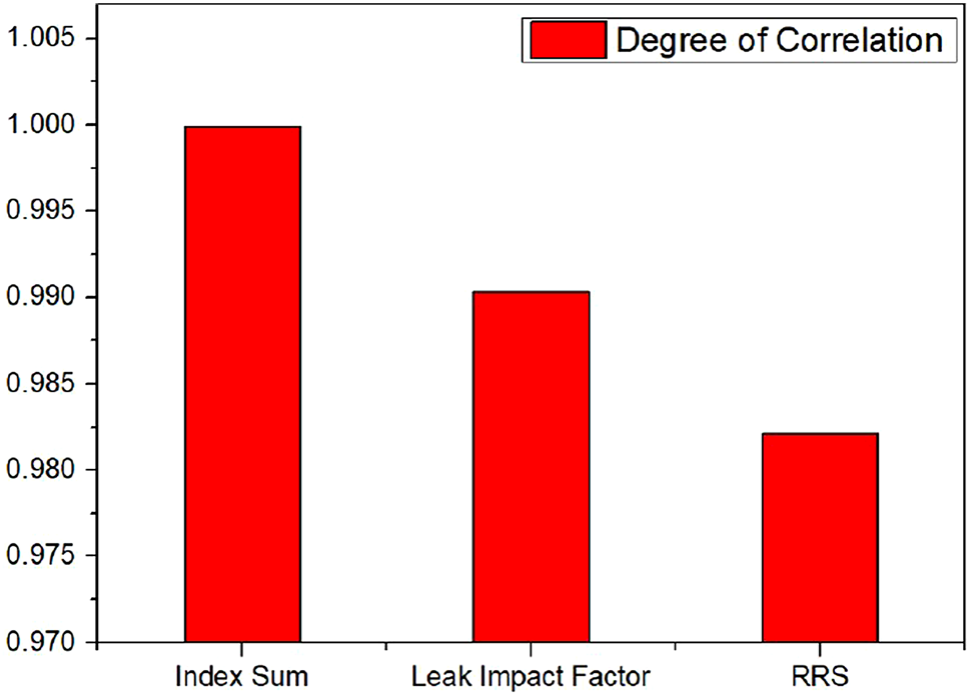
Correlation coefficient degree between qualitative and subtractive clustering method.

Figure 11 shows high correlation coefficient value ($\rho$ = 0.9999) for index sum, ($\rho$ = 0.9903) for leak impact factor, and ($\rho$ = 0.9821) for RRS, implies the effectiveness of using the TS fuzzy inference method based on subtractive clustering.

## Conclusion

Indexing pipeline risk assessment methodology is integrated with subtractive clustering fuzzy logic to deal with the uncertainty of the real-world conditions and to avoid the difficulties of constructing many rules. The computational complexity increases with the dimensions of the system variables because the number of rules increases exponentially as the number of system variables increases.

The proposed approach for pipeline risk assessment is demonstrated using a case study of a petroleum pipeline, with the results of the proposed model compared to the qualitative method. The pipeline is divided to seven sections and the risk assessment procedure is done for each section by both qualitative and proposed model. Results showed that the RRS values computed using the proposed model are consistent with those obtained using the qualitative method. The proposed model also had a high correlation and accuracy. The proposed model is evaluated using training RMSE, testing RMSE, and R^2^ of values 5.9653 × 10^–8^ and 7.35411 × 10^–8^ and 1 for index sum model, and 1.4065 and 8.7814 and 0.9601 for the leak impact factor model respectively. The proposed model is proven to be an efficient model for pipeline risk assessment using a fuzzy clustering approach. Hence, future work will be performed for risk assessment of several facilities within the offshore industry.

## Data Availability

The datasets generated during and/or analysed during the current study are available from the corresponding author on reasonable request.
